# Recruitment of Language-, Emotion- and Speech-Timing Associated Brain Regions for Expressing Emotional Prosody: Investigation of Functional Neuroanatomy with fMRI

**DOI:** 10.3389/fnhum.2016.00518

**Published:** 2016-10-18

**Authors:** Rachel L. C. Mitchell, Agnieszka Jazdzyk, Manuela Stets, Sonja A. Kotz

**Affiliations:** ^1^Centre for Affective Disorders, Institute of Psychiatry Psychology and Neuroscience, King's College LondonLondon, UK; ^2^Department of Psychology, Durham UniversityDurham, UK; ^3^Department of Psychology, University of EssexColchester, UK; ^4^Section of Neuropsychology and Psychopharmacology, Maastricht UniversityMaastricht, Netherlands

**Keywords:** emotional prosody, prosody expression, speech, social cognition, fMRI

## Abstract

We aimed to progress understanding of prosodic emotion expression by establishing brain regions active when expressing specific emotions, those activated irrespective of the target emotion, and those whose activation intensity varied depending on individual performance. BOLD contrast data were acquired whilst participants spoke non-sense words in happy, angry or neutral tones, or performed jaw-movements. Emotion-specific analyses demonstrated that when expressing angry prosody, activated brain regions included the inferior frontal and superior temporal gyri, the insula, and the basal ganglia. When expressing happy prosody, the activated brain regions also included the superior temporal gyrus, insula, and basal ganglia, with additional activation in the anterior cingulate. Conjunction analysis confirmed that the superior temporal gyrus and basal ganglia were activated regardless of the specific emotion concerned. Nevertheless, disjunctive comparisons between the expression of angry and happy prosody established that anterior cingulate activity was significantly higher for angry prosody than for happy prosody production. Degree of inferior frontal gyrus activity correlated with the ability to express the target emotion through prosody. We conclude that expressing prosodic emotions (vs. neutral intonation) requires generic brain regions involved in comprehending numerous aspects of language, emotion-related processes such as experiencing emotions, and in the time-critical integration of speech information.

## Introduction

In the study of social cognition, increasing efforts have been invested into learning more about how we transmit our communicative intent and alert other people as to our mental or emotional state of mind. Prosody is one channel by which we can express such emotion cues. By varying non-verbal features of speech such as pitch, duration, amplitude, voice quality, and spectral properties (Ross, 2010), we can alter our tone of voice, and change the emotion conveyed. Beyond automatic and true reflections of our emotional state, conscious modulation of emotional prosody may also be one of the most common emotion regulation strategies, with people frequently concealing or strategically posing their prosodic emotion cues in everyday interactions (Laukka et al., 2011). In parallel, neuroscientists have sought to uncover the brain mechanisms that underpin the transmission of these signals. Because of the lag behind facial emotion research, its multiple functions (e.g., linguistic, attitudinal, motivational, affective), and multiple phonetic cues (e.g., pitch, duration, amplitude), the neural substrate of emotional prosody expression is less well-characterized (Gandour, 2000).

## Concordance with early lesion-based models of prosodic expression

In the 1970s and 1980s, a series of papers reporting lesion studies associated damage to the right hemisphere homolog of Broca's area (Brodman's areas 44 and 45) with impaired ability to produce emotional prosody, whilst damage to the posterior temporal region appeared to be associated with an inability to comprehend emotional prosody (Ross, 1981; Ross et al., 1981; Gorelick and Ross, 1987). Thus, it seemed that the organization of prosodic functions in the right hemisphere mirrored that of propositional language functions in the left hemisphere. Primarily because of speech-related movement confounds which can induce signal changes independent of those related to neuronal activation (Gracco et al., 2005), direct functional magnetic resonance imaging (fMRI) literature on the expression of emotional prosody is limited. Sparse auditory sequences have gone some way to ameliorating these movement confounds though (Hall et al., 1999), and neuroimaging studies of prosodic emotion expression are starting to emerge.

In one study, participants produced sentence-like sequences of five syllables (e.g., dadadadada) in various tones of voice, and when the expression of emotional intonation was compared to use of a monotonous voice, activation was observed in the right inferior frontal gyrus, as predicted by the lesion study model (Mayer et al., 2002). However, in another study using similar methodology but comparing prosodic emotion expression to rest, the active region was the anterior right superior temporal gyrus instead (Dogil et al., 2002). More recently, inferior frontal gyrus activity has been detected during the preparation and execution of emotional prosody expression (Pichon and Kell, 2013), although its degree of activation differed between the two phases of the expression process. Similarly, in another recent study of emotional prosody expression the inferior frontal gyrus was in fact the only region whose activation depended on both the emotion vocalized and the specific expression task (repetition vs. evoked) (Frühholz et al., 2014). Thus, from the evidence available so far, inferior frontal gyrus activation is not consistent. Where similar methodology is employed across studies, one possibility is that its activation might relate to the composition of the participant sample.

Another shift in thinking in recent years concerns the relationship between the neural systems that mediate the expression and comprehension of speech. For propositional language, a “mosaic” type view of its organization in the brain has emerged, in which there is partial overlap between the brain regions that subserve its comprehension and expression (Gandour, 2000; Hickok and Poeppel, 2004). Hints are now emerging that this may also be true for prosody. In the main study of relevance, overlapping involvement in the expression and comprehension of emotional prosody was demonstrated in several brain regions, including the left inferior frontal gyrus, left middle cingulate gyrus, right caudate, and right thalamus (Aziz-Zadeh et al., 2010). Thus, further studies of emotional prosody expression perhaps need to be vigilant for additional signs that there is merit to this organizational overlap.

## The involvement of sub-cortical brain regions in prosodic expression

Whilst it was concluded from one of the early studies that prosody expression is mediated exclusively by neocortical brain structures (Dogil et al., 2002), elsewhere lesion data suggests its expression may also necessitate subcortical brain regions such as the basal ganglia. Basal ganglia damage has been observed to lead to both a restricted pitch contour with less variability in pause duration (Blonder et al., 1995), and foreign accent syndrome, a condition in which abnormal prosody articulation leads to the perception of a foreign-like accent (Carbary et al., 2000). The basal ganglia have also been the most frequently damaged structure in larger samples of aprosodic patients (Cancelliere and Kertesz, 1990). This role of the basal ganglia in prosody expression likely reflects its involvement in the timing-related processes which can be used to establish basic routines that advance more sophisticated behavior e.g., formulating specific emotional intonation (Kotz and Schwartze, 2010). However, basal ganglia involvement in emotional prosody expression may not only be associated with preparing for the expression of emotional prosody as suggested by one recent fMRI study (Pichon and Kell, 2013). It may also integrate and maintain dynamically changing speech information such as speech rate, pitch, or amplitude (intensity) variations into coherent emotional gestalts (Paulmann and Pell, 2010), which perhaps better describes the execution of emotional prosody expression. Activation of the basal ganglia was detected in a recent neuroimaging study of the evocation of emotional prosody expression, but that study focused exclusively on the expression of angry prosody (Frühholz et al., 2014).

## Aims and hypotheses

Using methodological refinements, we aimed to expand recent progress in delineating the functional neuroanatomy of prosodic emotion expression. Our first adaptation concerned the conditions to which prosodic emotion expression is compared. We included not just a neutral condition but also a covert speech condition with jaw movement, to evaluate the functional neuroanatomy associated with expressing neutral prosody, i.e., a non-emotional prosodic contour.

Secondly, based on recent meta-analyses and reviews of emotion-specific differential emotion processing (Phan et al., 2002; Chakrabarti et al., 2006; Fusar-Poli et al., 2009; Vytal and Hamann, 2010; Lee and Siegle, 2012), we aimed to determine whether the brain mechanisms behind prosodic emotion expression differed as a function of specific positive and negative valence exemplars. Reliable emotion-specific effects have not yet been agreed for the brain networks mediating comprehension of prosodic emotions, with some researchers suggesting that there are separate networks (Ethofer et al., 2009; Kotz et al., 2013b), and others suggesting that there are not (Wildgruber et al., 2005). One possibility is that the brain regions involved in expressing specific emotions are similar to those reported for perceiving that emotion. For prosody, the early indications are that processing other people's happiness cues involves the middle temporal gyrus and inferior frontal gyrus (Johnstone et al., 2006). Networks associated with the perception of angry prosody have been studied in more detail, and prominent regions include the anterior cingulate, inferior frontal gyrus/orbitofrontal cortex, middle frontal gyrus, insula, thalamus, amygdala, superior temporal sulcus, fusiform gyrus, supplementary motor area (Grandjean et al., 2005; Sander et al., 2005; Johnstone et al., 2006; Ethofer et al., 2008; Frühholz and Grandjean, 2012). Whilst one study has identified the specific regions associated with expressing neutral prosody, the results may reflect a lack of control for motor movement (Dogil et al., 2002). It might also be possible that the brain regions for expressing angry prosody bear some similarity to those involved in the experience of being or feeling angry, and similar for happiness (Lee and Siegle, 2012). One might then expect the expression of angry prosody to involve brain regions previously associated with feeling angry, such as the medial prefrontal gyrus, insula, and cingulate cortex (Denson et al., 2009), and the expression of happy prosody to involve brain regions previously associated with feeling happy, e.g., the basal ganglia (Phan et al., 2002), and possibly cortical regions in the forebrain and limbic system (Kringelbach and Berridge, 2009).

Our final aim was to determine the between-person variability of the neural system for expressing emotional prosody, i.e., to determine the parts of the system subject to individual differences. We probed this question by examining in which brain regions did activation levels covary with successful expression of prosodic emotions? Do individuals who are better at expressing prosodic emotions recruit brain regions that those not so good at expressing prosodic emotions do not? Individual differences in the ability to express emotional prosody have long been recognized at the behavioral level (Cohen et al., 2010), so what is the mechanism by which these effects occur (Blakemore and Frith, 2004)? In addressing this final aim, we noted that to date, few studies have examined individual differences in socio-cognitive skills and linked these to underlying neural function (Corden et al., 2006). As to which brain regions might display such a relationship, we explored the possibility that inconsistent inferior frontal gyrus activation between studies might be explained by between-study differences in the abilities of the samples of healthy young adults recruited. Individual differences in ability have already been shown to influence the brain regions detected in neuroimaging studies of prosodic emotion comprehension (Sander et al., 2003; Schirmer et al., 2008; Aziz-Zadeh et al., 2010; Kreifelts et al., 2010; Jacob et al., 2014). Based on the association between basal ganglia impairment and a monotone voice with low prosodic expressivity (Martens et al., 2011), we also tested whether activity in this region correlates with the ability to transmit appropriate emotional prosody.

## Materials and methods

### Participants

Twenty-seven healthy young adults (14 females, 13 males) were recruited by email and word of mouth from amongst staff and students at Durham University. This end sample comprised a mean age of 21.5 years (± 3.89). Besides the target participant age range of 18–35 years, a further inclusion criterion was that participants must be native English speakers given the subtle nature of the task. All reported themselves as being right-handed, which was subsequently confirmed through scores >40 across all participants on the Edinburgh Handedness Inventory (Oldfield, 1971). Across the end sample, the mean number of years of formal education was 15.7 years (± 2.01). Upon initial contact, exclusion criteria applied to those who volunteered included self-reports of history of uncorrected hearing deficits, history of psychiatric or neurological illness, significant head injuries or long periods of unconsciousness, history of alcohol or drug abuse, and MRI contraindications (all self-report). As background assessments to characterize our group of participants, Beck's Depression Inventory (BDI; Beck and Steer, 1987) and the Positive and Negative Affect Schedule (PANAS; Watson et al., 1988) were administered. Mean BDI was 4.5 (± 5.65), indicating that the group displayed only minimal symptoms of depression. In keeping with relevant normative data, the positive affect of our participants was 38.1 (± 6.75), and the negative affect was 17.1(± 6.07; Crawford and Henry, 2004). Participants were paid a flat fee of £25 for their participation, covering their time, travel and inconvenience.

The study described was performed in accordance with the declaration of Helsinki (Rits, 1964), and the British Psychological Society guidelines on ethics and standards (http://www.bps.org.uk/what-we-do/ethics-standards/ethics-standards). Approval for its conduct was given by the Ethics Advisory Sub-Committee in the Department of Psychology, Durham University, and written informed consent was obtained from all those who participated.

### Experimental task

The event-related expression task administered during fMRI comprised four conditions: Happy intonation [as the only widely accepted positive “basic” or “primary” emotion (Ekman, 1992)], angry intonation (as a negative “basic” emotion), neutral intonation, and jaw movement. Thus, like the Aziz-Zadeh et al. study, our design was balanced across positive and negative emotion trials, in contrast to the methodology of Pichon and Kell that sacrificed balance between positive and negative emotions for generalizability across a wider range of emotions (Aziz-Zadeh et al., 2010; Pichon and Kell, 2013). The stimuli in these conditions were pronounceable non-sense words (see Supplementary Materials; Kotz, 2001), derived from real neutral valence words by substituting a single letter of the original real word (e.g., normal → “narmal”). Rendition of non-sense words enters the speech production process low enough to eliminate higher-level linguistic processing (Mayer, 1999), and therefore allowed us to exclude potentially confounding semantic connotations as might theoretically be incurred in studies without this feature. Participants were presented with three randomly selected non-sense words at a time arranged vertically and centrally onscreen, with an emotion prompt in emboldened capital letters at the top of the screen. At the start of the task, they were instructed that they would be prompted which word to say and when to speak it. As each of the three non-sense words in turn changed from black non-underlined font to red underlined font with a star next to it, participants were instructed to say that word out loud in the tone specified at the top of the screen. Although fully debriefed after the study, during the fMRI session, participants were unaware that their online vocalizations were not recorded. All text was displayed in Calibri point 60, using E-prime experiment generation software v2 (Psychology Software Tools; Sharpsburg, PA, USA). Visualization was achieved via display on an LCD screen mounted on a tripod at the rear of the scanner (Cambridge Research Systems; Rochester, Kent, UK), and standard head-coil mounted mirrors.

To probe valence-dependence, we used anger as the negative emotion rather than sadness as used by Aziz-Zadeh et al. (2010), based on findings that anger is a more easily recognizable negative emotion than sadness (Paulmann et al., 2008). From the four available prompts (angry, happy, neutral, and jaw), the emotion cue displayed was randomized through the paradigm. When participants saw the prompt JAW rather than angry/happy/neutral, they were asked to move their jaw and tongue as if saying the word out loud, but not actually say it out loud (Dhanjal et al., 2008). This jaw condition better controlled for speech movement-related activation than a simple rest condition would have done, and enabled us to separate movement induced confounds from activations that truly relate to the external vocalization of prosody. The inclusion of the neutral condition further allowed us to distinguish those brain regions that specifically related to conveying emotion (happy/angry) through prosody rather than producing prosody in general (neutral). The design was such that the speaking of one non-sense word was linked to each brain volume collected. All three of the non-sense words were to be spoken with the same specified tone before the task moved on to the next triplet, to increase detection power for the neural response associated with each condition (Narain et al., 2003). In total, there were 80 triplets, i.e., 240 individual words or trials.

### Listener ratings of prosodic emotion expression

In this preliminary study, MRI participants expressing emotion cues through tone of voice were recorded performing this task offline in a separate behavioral assessment. Importantly, the prosodic emotion expression task used in this behavioral assessment was identical in structure and timings to that used in the MRI assessment. Whilst performing this task, participants' audio output was recorded on an Edirol R4 portable recorder and wave editor (Roland Corporation; California, USA), in conjunction with an Edirol CS-50 Stereo Shotgun microphone. Half the participants were tested for the behavioral assessment before the day of their MRI assessment, whilst the others were tested on a date after their MRI assessment. One MRI participant did not attend their behavioral assessment session. The mean gap between the MRI and behavioral assessments was 11.3 (± 4.03) days. The behavioral and MRI assessments were run separately, because even with the sparse acquisition sequence described below, some artifacts in the functional images caused by movement of the articulators (head, lips, tongue, and larynx) and head remain (Elliott et al., 1999). Indeed, offline recording prior to subsequent fMRI has been the method most often used to assess participants' ability to express prosodic emotions in other studies (Mayer, 1999; Mayer et al., 2002; Pichon and Kell, 2013). In accordance with the offline recording strategy, it has been shown that the conditions typically experienced whilst being scanned do not seem to influence prosody generation (Mayer, 1999).

To evaluate the MRI participants' recordings, a further 52 healthy young adults were recruited from the research panel of psychology undergraduates (M:F 3:49) at Durham University. The mean age of this group of listeners was 19.1 (± 0.78) years, their mean weekly alcohol consumption was 7.0 (± 3.73) UK units, and their mean number of years' education was 14.4 (± 0.90). To screen for listeners whose hearing sensitivity might be impaired, a Kamplex KS8 audiometer was used to determine hearing sensitivity loss relative to British Standard norms BS EN 60645, and BS EN ISO 389. Tones were presented at central pure-tone audiometry frequencies, namely 500, 1, and 2 kHz. The pure tone average was derived by computing mean hearing sensitivity across both ears and all frequencies. The cut-off point for screening purposes was set at the clinically normal limit of < 25 dB hearing level (HL) (Leigh-Paffenroth and Elangovan, 2011), but no listeners had to be excluded on this basis.

A pair of listeners listened to the recording of each MRI participant made in the behavioral assessment. Listeners were instructed to listen to each triplet of non-sense words and select from the three-alternative forced choice options of happiness, anger and neutrality, their subjective judgment of which emotion they thought was conveyed by speaker intonation. The influence of ambient noise on this listening task was ameliorated by presenting the audio recordings via noise cancelation headphones (Quiet Comfort 3; Bose Corporation; Framingham, MA). In scoring the ability of MRI participants to convey emotions through prosody, each non-sense word was only scored as correct if both listeners agreed on the emotion (i.e., 100% concordance), *and* that emotion was what the MRI participant had been instructed to use. After each pair of listeners had rated all their assigned audio clips, Cohen's kappa was used to determine if there was agreement between the two listeners' judgments of the emotion conveyed. These analyses determined that across the listener pairs for the set of MRI participant recordings, the mean agreement within each pair was moderate, κ = 0.498 (± 0.049 s.e.).

To further assess the distinctiveness of the happy, angry and neutral styles of emotional prosody expression, the acoustic correlates of the offline speech recordings were analyzed using the auditory-processing software “Praat” (Boersma, 2001). By this endeavor, the features extracted for analysis of each prosodic emotion type included mean fundamental frequency, fundamental frequency standard deviation and fundamental frequency range to index pitch; mean amplitude and amplitude range to index intensity; and duration. Following feature extraction with PRAAT, the mean values for each index were compared across prosodic emotion types with one-way ANOVAs.

### MRI data acquisition

Given that speaking involves movement and that fMRI is susceptible to motion and volume-change artifacts, previous fMRI studies of language and speech production often used “inner” or “covert” speech or whispering (Dogil et al., 2002; Gracco et al., 2005). We implemented a sparse audio neuroimaging sequence, because their advent has much improved the ability to study (overt) speech production functions (Dhanjal et al., 2008; Simmonds et al., 2011). In these temporally sparse imaging protocols (Hall et al., 1999), relatively long silent pauses are included between volume acquisitions, and it is during these pauses that stimuli are presented making it unlikely that stimulus-induced neural responses are obscured by scanner-noise-induced neural responses (Moelker and Pattynama, 2003; Blackman and Hall, 2011; Liem et al., 2012), as might theoretically have occurred in one recent fMRI study of emotional prosody expression (Pichon and Kell, 2013). Data were acquired on a 3T MRI scanner with 32 channel head coil (Siemens TRIO, Siemens Medical Solutions, Erlangen, Germany) at the Durham University and South Tees NHS Trust MRI facility (U.K.). The sequence also employed Siemens' parallel acquisition technique “iPAT” (Sodickson and Manning, 1997), deployed with generalized auto calibrating partially parallel acquisition acceleration factor 2 (GRAPPA) (Griswold et al., 2002), to further reduce the opportunity for motion artifacts (Glockner et al., 2005). Instructional measures taken to minimize motion artifacts included the explicit direction that participants should hold their head as still as possible at all times, and the use of foam padding between a participant's head and the head coil itself.

In the transverse plane parallel to anterior-posterior commissure line, we acquired blood oxygenation level dependent (BOLD) contrast images with a non-interleaved MRI EPI sequence with 30 ms TE, and an 8 s repetition time (TR) in which a 1.51 s acquisition time (TA) was followed by 6.49 s silence. In all, 240 brain volumes were collected. To capture BOLD responses over the whole cerebrum, twenty eight-4 mm slices alternated with a 5 mm gap, over a 192 mm field of view with 64 × 64 matrix and 90° flip angle. The first true radio frequency pulse generated by the scanner triggered E-prime to synchronize stimuli presentation with data collection. To maintain synchronicity, the start of subsequent trials was also triggered by each new pulse. To raise the effective sampling rate (Josephs and Henson, 1999), within each 8 s TR the speaking cue was jittered randomly between 2 and 3 s after the start of volume acquisition, i.e., 5, 6 s before the next volume was acquired (Belin et al., 1999). The analyses described below therefore specifically focused on the execution of emotional prosody expression. To facilitate individual localization of active brain regions, anatomical data were collected with a Magnetization Prepared RApid Gradient Echo single-shot T1-weighted sequence (Mugler and Brookeman, 1990), in the same orientation as the functional data, with one hundred and ninety two-9 mm slices alternating with a 45 mm gap. The sequence incorporated a TR of 1900 ms a TE of 2.32 ms, and field of view 230 mm. As for the functional sequence, the anatomical sequence employed “iPAT,” with GRAPPA factor 2.

### Functional MRI data analyses

The first four scans were discarded whilst the MR signal reached a steady state. Neuroimaging data were then analyzed with SPM8 (www.fil.ion.ucl.ac.uk/spm/software/spm8). In initial pre-processing, images were realigned using the first image as a reference, using the SPM realignment function. Despite the movement involved in overt speech, no participant displayed more than 0.5 mm translation or 0.5 degrees rotation in any plane during the scans, thus no data were excluded due to potentially confounding effects of excessive movement. Images were then normalized into a standard stereotactic space to account for neuroanatomic variability, using the Montreal Neurologic Institute ICBM152 brain template in SPM, and applying spatial normalization parameters generated by prior segmentation of tissue classes with SPM. Last in pre-processing, the images were smoothed using an isotropic Gaussian kernel filter of 8 mm full-width half-maximum, using the SPM smoothing function.

In the first level analyses, the pre-processed data were analyzed in an event-related manner. In line with established thinking, the design matrix did not convolve the design with a haemodynamic response function as implemented by Pichon and Kell (2013), but rather a finite impulse response (FIR) model was implemented (Gaab et al., 2007a,b). This model-free approach is known to account for additional sources of variance and unusual shaped responses not well captured by a single haemodynamic response function (Henson, 2004). Once constructed, the FIR models were then estimated, to yield one mean contrast image per participant, using a 128-s high pass filter for each model. For each individual MRI participant, the search volume for the first-level analyses was constrained by the implementation of an explicit (“within-brain”) mask derived from the combination of each MRI participant's gray and white matter image generated from the segmentation phase of pre-processing. This strategy reduced the potential for false positives due to chance alone—the “multiple comparisons problem,” and helped to limit seemingly significant activations to voxels within the brain rather than those covering cerebrospinal fluid or those that lay outside the brain.

At the second level, random effects analyses were performed, to ascertain common patterns of activation across the participants, and enable inferences about population-wide effects. To examine the brain regions associated with expressing prosody of an emotional nature, regional brain activity patterns during the expression of happy and angry prosody were each contrasted separately against the regional brain activity associated with expressing neutral prosody. To examine the brain regions associated with expressing a prosodic contour that did not convey emotion, the pattern of regional brain activity observed during the expression of neutral prosody was compared against that observed during the jaw movement condition. To establish how the patterns of regional brain activity during the expression of angry and happy prosody differed from each other, we examined the brain regions in which the neural response during angry prosody expression was significantly greater than that during happy prosody expression, and vice versa. In these latter analyses, any effect of differences in performance accuracy between the expression of angry and happy prosody was excluded by including a performance accuracy covariate in the model, performance accuracy being operationalized as the percentage of trials for which both raters agreed that each MRI participant had indeed expressed each emotion. Common regions of activation associated with the expression of both happy AND angry prosody were examined through the implementation of a “conjunction null” test in SPM. To probe individual differences in the neural system responsible for expressing prosodic emotions, a covariate for performance accuracy on the offline behavioral assessment was fed into a second-level whole-brain analysis contrasting those brain regions associated with the expression of angry and happy prosody against those associated with the expression of neutral prosodic contours. In this analysis, it was the brain regions whose activity correlated with performance accuracy that was of interest, perceived performance accuracy being collated across the expression of the two emotional types of prosody.

Activations were thresholded at *p* < 0.05, corrected for multiple comparisons with the Family Wise Error adjustment based on random field theory (Brett et al., 2003). The non-linear transforms in the Yale BioImage Suite MNI to Talairach Coordinate Converter (www.bioimagesuite.org/Mni2Tal/) (Lacadie et al., 2008) converted “ICBM152” MNI template coordinates to approximate Talairach and Tournoux coordinates (Talairach and Tournoux, 1988), enabling use of the Talairach and Tournoux atlas system for identifying regions of statistically significant response. Individual regions of activation were identified and labeled using the Talairach Daemon applet (http://www.talairach.org/applet.html) (Lancaster et al., 1997, 2000).

## Results

### Behavioral performance

The analyses reported in this section were all performed using IBM SPSS Statistics for Windows, Version 22.0 (Armonk, NY: IBM Corp.). The main index of behavioral performance was the offline evaluation of MRI participants' ability to express a given emotional tone i.e., happy, angry, or neutral. The correct agreement by both raters that the given tone was indeed reflected in the tone of voice they heard varied was emotion-dependent, from 66.3% (± s.e. 5.13) of the time for happiness, through 62.6% (± s.e. 4.46) for neutral, to 53.1% (± s.e. 5.07) for anger. These figures are comparable to previous reports on the correct attribution of prosodic cues to specific emotion categories (averaged across cold and hot anger for angry expressions) (Banse and Scherer, 1996; Johnstone and Scherer, 2000). The ANOVA suggested a main effect of emotion in these performance data [*F*_(2, 50)_ = 3.95, *p* < 0.05, η^2^ = 0.096]. However, for all three emotion conditions, the perceived expression accuracy was over 4 × greater than the 1-in-9 level of correct agreement expected by chance, a difference that was highly significant according to one-sample *t*-test analyses [happy: *t*_(25)_ = 10.75, *p* < 0.001, *d* = 2.108; neutral: *t*_(25)_ = 11.55, *p* < 0.001, *d* = 1.776; anger: *t*_(25)_ = 8.29, *p* < 0.001, *d* = 1.625]. Further, interrogation of the performance data determined that for each of the three conditions (happy, angry, and neutral), no outliers were detected for the percentage of correct rater1-rater2 agreement amongst the group of MRI participant recordings. Specifically, none of the figures for the rater pair cases fell more than 1.5 × the inter-quartile range above the third quartile or below the first quartile.

The analyses of the acoustic correlates of each emotional prosody style further supported the interpretation that participants were able to produce perceptually distinguishable prosody, i.e., they were able to adequately modulate the acoustics features of their speech to express emotions. These acoustic correlate data are summarized in Table 1. A significant main effect of emotion was observed for all acoustic indices (*p* < 0.05 or lower). Worthy of note, follow-up paired *t*-test analyses revealed that happy prosody was of higher pitch than either angry or neutral prosody (*p* < 0.001 for both) (Pierre-Yves, 2003; Fragopanagos and Taylor, 2005; Scherer, 2013; Ooi et al., 2014). Speakers demonstrated greater pitch modulation (F0 s.d.) for both angry and happy prosody than for a monotone “neutral” intonation (*p* < 0.05 for both) (Pierre-Yves, 2003; Fragopanagos and Taylor, 2005; Pell et al., 2009). The mean amplitude of angry prosody was, as might be expected, greater than that of neutral prosody (*p* < 0.001) (Ververidis and Kotropoulos, 2006). Speakers also demonstrated greater amplitude modulation (amplitude range) for both angry and happy prosody than for “neutral” intonation (*p* < 0.001 for both) (Scherer, 2013). These patterns of effects are consistent with prior literature (Scherer, 1986, 2003; Banse and Scherer, 1996; Juslin and Laukka, 2003; Juslin and Scherer, 2005).

**Table 1:** **The acoustic correlates of emotional prosody expression**.

**Acoustic feature**	**Emotional prosody type**			**Comparative analyses**
	**Angry**	**Happy**	**Neutral**	
Mean F0 (Hz)	174.51 (± 41.72)	213.72 (± 55.32)	178.53 (± 44.31)	*F*_(2, 52)_ = 27.773, *p* < 0.001
F0 s.d. (Hz)	23.81 (± 8.22)	30.65 (± 11.03)	24.55 (± 11.89)	*F*_(2, 52)_ = 4.707, *p* < 0.05
F0 range (Hz)	68.08 (± 22.37)	89.73 (± 29.86)	73.59 (± 29.26)	*F*_(2, 52)_ = 6.330, *p* < 0.005
Mean amplitude (dB)	61.28 (± 5.12)	61.25 (± 5.20)	58.42 (± 5.40)	*F*_(2, 52)_ = 16.843, *p* < 0.001
Amplitude range (dB)	41.75 (± 5.49)	41.42 (± 4.33)	38.36 (± 4.16)	*F*_(2, 52)_ = 17.775, *p* < 0.001
Duration (s)	0.57 (±.09)	0.55 (±.08)	0.54 (±.08)	*F*_(2, 52)_ = 4.306, *p* < 0.05

*Mean pitch, intensity and duration statistics (± s.d.) for each prosodic emotion style, from the offline speech recordings of study participants*.

### fMRI data

ANOVA analyses of the translational estimated movement parameters (derived during the realignment stage of the SPM pre-processing pipeline) with SPSS demonstrated that there were no differences between the angry, happy, jaw, and neutral conditions in the degree of movement in the x, y, and z planes. The main effects of emotion condition and plane were not significant [(*F*_(3, 78)_ = 0.51, *p* = 0.68, η^2^ = 0.019) and (*F*_(2, 52)_ = 0.83, *p* = 0.44, η^2^ = 0.031) respectively], and neither was the interaction between them [*F*_(6, 156)_ = 0.35, *p* = 0.91, η^2^ = 0.013]. Similarly, analyses of the rotational estimated movement parameters did not find any evidence of significant differences between the angry, happy, jaw, and neutral conditions in the degree of rotation about the x, y, and z planes. Again, the main effects of emotion condition and plane were not significant [(*F*_(3, 78)_ = 0.65, *p* = 0.58, η^2^ = 0.025] and [*F*_(2, 52)_ = 0.06, *p* = 0.95, η^2^ = 0.002) respectively], and the interaction between them was not significant either [*F*_(6, 156)_ = 0.79, *p* = 0.58, η^2^ = 0.030).

**Figure 1:**
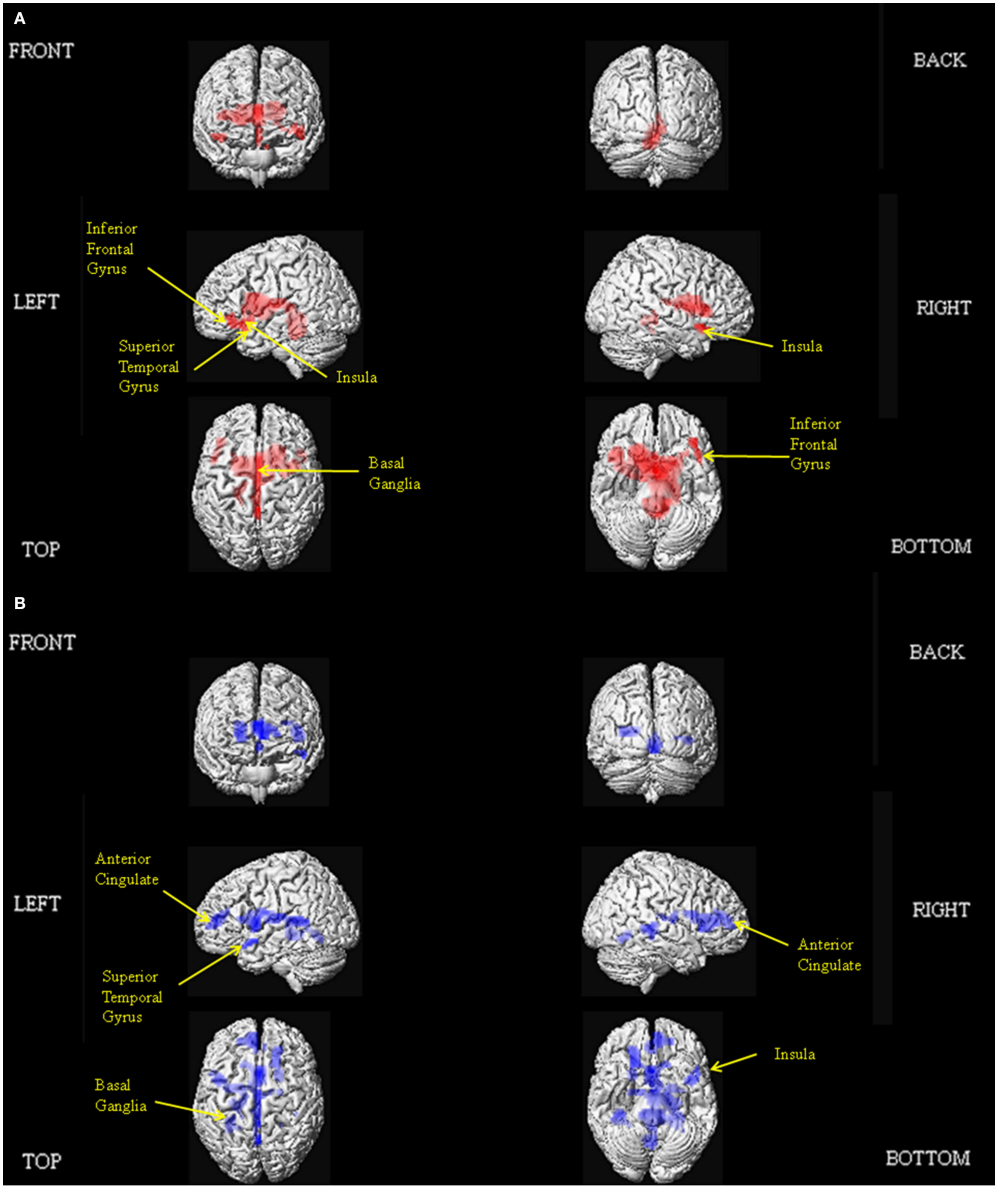
**Depiction of the brain regions activated when expressing anger (A), and happiness (B) through prosody (relative to neutrality), displayed on a rendered brain derived from the Montreal Neurological Institute Ch2bet.nii image supplied with the MRIcroN software (http://www.mccauslandcenter.sc.edu/mricro/mricron/index.html)**. Regions of activation on the external surface of the cortex appear brighter and more intense, whereas regions deeper in the cortex are displayed in less intense, more transparent shades. Images are thresholded at *P*_FWE_ <0.05 with a 10 voxel spatial contiguity threshold.

**Figure 2:**
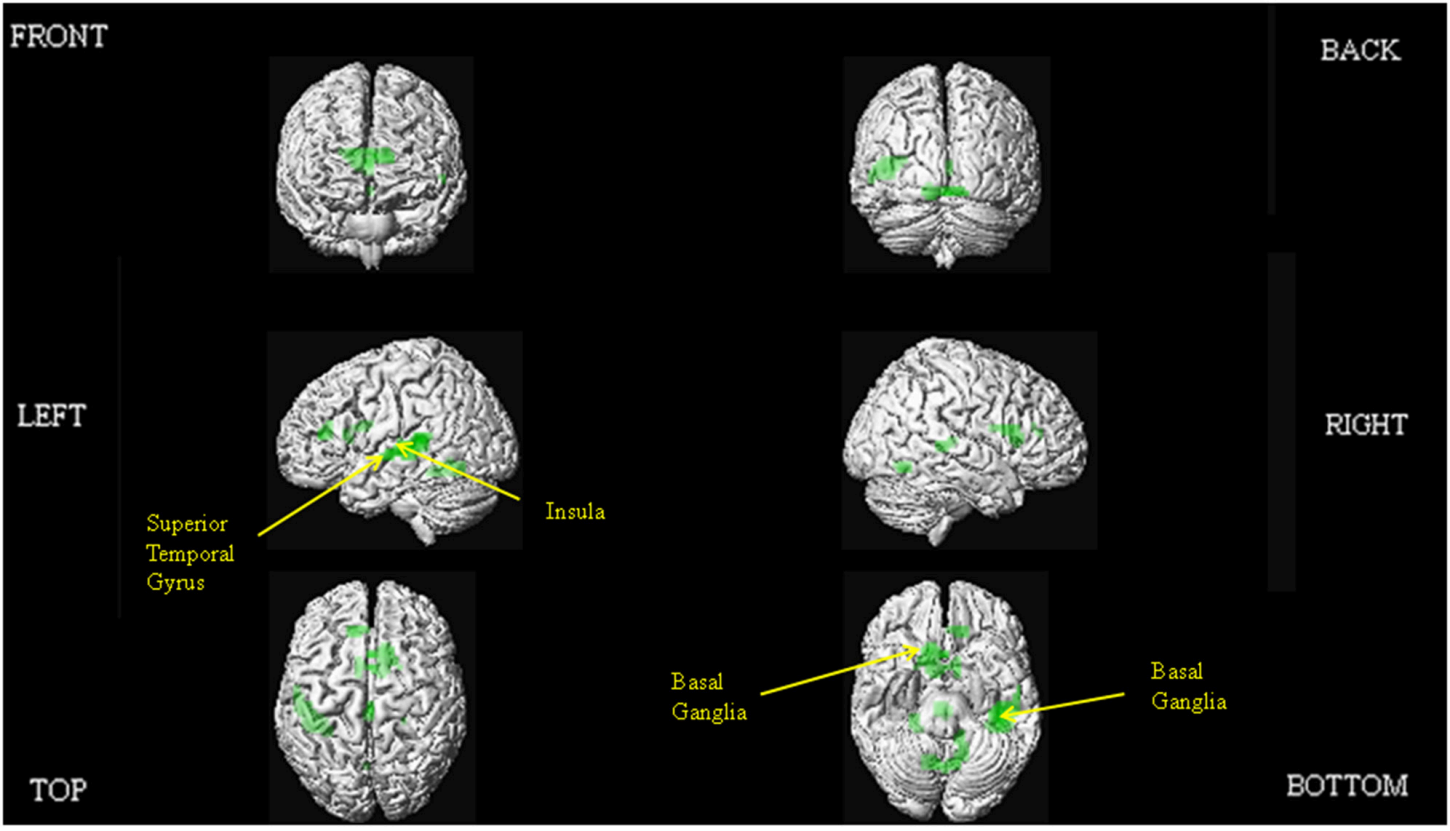
**Depiction of the brain regions activated when expressing neutrality through prosody (relative to jaw movement), displayed on a rendered brain derived from the Montreal Neurological Institute Ch2bet.nii image supplied with the MRIcroN software (http://www.mccauslandcenter.sc.edu/mricro/mricron/index.html)**. Regions of activation on the external surface of the cortex appear brighter and more intense, whereas regions deeper in the cortex are displayed in less intense, more transparent shades. Images are thresholded at *P*_FWE_ <0.05 with a 10 voxel spatial contiguity threshold.

**Figure 3:**
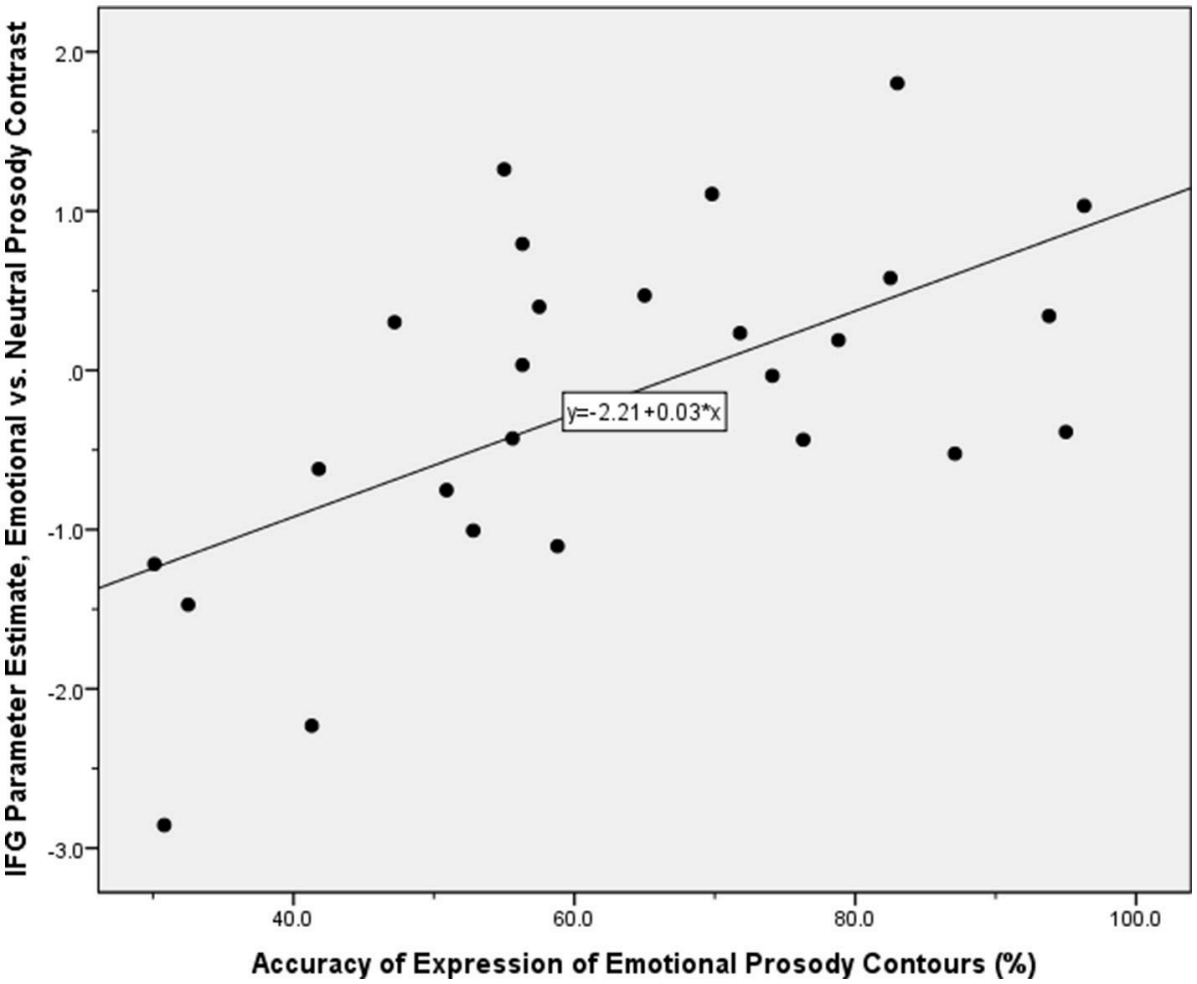
**Scatter plot illustrating the correlation between the parameter estimate for the contrast of emotional vs. neutral prosody expression in the inferior frontal gyrus, and the offline index of the accuracy with which participants expressed emotional prosodic contours**. Application of the Kolmogorov-Smirnov test for normality indicated that these performance accuracy data were normally distributed: *d*_(26)_ = 0.091, *p* > 0.05.

The results of our main analyses of the fMRI data are presented in Table 2, Figures 1–**3**. Relative to brain regions associated with the expression of neutral prosody, the key regions associated with the expression of angry intonation included the inferior frontal gyrus, superior temporal gyrus, basal ganglia, and insula (Table 2, Figure 1A). The expression of happiness through intonation also recruited the superior temporal gyrus, basal ganglia, and insula, with the additional involvement of parts of the anterior cingulate (Table 2, Figure 1B). The expression of a neutral prosodic contour saw activation in the basal ganglia, anterior cingulate, superior temporal gyrus, and insula again (Table 2, Figure 2). The conjunction of areas activated by the angry vs. neutral and happy vs. neutral contrasts, formally revealed overlapping activation in the superior temporal gyrus and basal ganglia (Table 2). Direct comparison between angry and happy prosody ascertained that expressing angry prosody resulted in greater activation in parts of the basal ganglia and insula than when expressing happy prosody, whilst expressing happy prosody resulted in greater activation of the anterior cingulate and other parts of the insula and basal ganglia than when expressing angry prosody (Table 2).

**Table 2:** **The expression of emotions through prosody: Stereotactic peak coordinates in contrasts of interest**.

**Brain region**	**Brodmann area**	**Hemisphere**	***T*-value**	**Stereotactic coordinates**
**ANGRY PROSODY VS. NEUTRAL PROSODY**				
Inferior frontal gyrus	47	L	6.56	–41 28 –4
“ “	47	R	5.20	40 15 –8
Superior temporal gyrus	38	L	4.83	–44 12 –9
Insula	13	L	5.86	–46 7 0
“ “	13	R	4.88	45 7 –5
Basal ganglia (caudate)		M	8.74	0 3 14
Thalamus		L	10.05	–2 –11 11
“ “		L	8.35	–3 –6 4
**HAPPY PROSODY VS. NEUTRAL PROSODY**				
Anterior cingulate	32	L	5.30	–6 43 11
“ “	32	L	5.30	–6 45 2
“ “	32	R	4.82	2 38 14
Superior temporal gyrus	38	L	5.22	–49 6 –9
Insula	13	L	6.21	–40 2 11
Basal ganglia (caudate)		L	8.67	–23 –42 15
“ “		M	8.50	0 9 16
“ “		R	6.79	17 23 3
“ “		R	5.61	17 21 13
“ “		R	5.34	20 6 19
“ “		R	5.38	35 –31 5
Thalamus		M	8.69	0 –14 12
**NEUTRAL PROSODY VS. JAW MOVEMENT**				
Anterior cingulate	32	L	4.64	–12 30 15
Superior temporal gyrus	22	L	5.44	–51 –14 4
Insula	13	L	7.93	–37 –26 4
Parahippocampal gyrus	30	R	5.53	20 –37 –1
Basal ganglia (caudate)		L	6.96	–37 –35 –1
“ “		R	6.50	11 15 19
“ “		R	5.58	8 18 11
“ “		R	5.01	8 3 19
Thalamus		R	6.50	3 –22 7
“ “		R	5.70	20 –28 10
**HAPPY PROSODY VS. ANGRY PROSODY**				
Anterior cingulate	32	L	9.93	–18 40 6
“ “	32	R	9.31	14 35 12
“ “	32	L	7.97	–3 42 3
Insula	13	L	6.91	–29 –39 15
Insula (Claustrum)		R	5.62	32 –25 7
Basal ganglia (caudate)		L	4.44	–23 –42 10
Thalamus		R	6.11	20 –34 10
**ANGRY PROSODY VS. HAPPY PROSODY**				
Insula	13	L	4.66	–31 –25 13
Hippocampus		L	5.51	–31 –40 0
Basal ganglia (putamen)		L	8.61	–17 –2 11
Basal ganglia (caudate)		R	6.36	2 3 11
“ “		R	5.34	11 11 8
**CONJUNCTION OF AvN and HvN**				
Superior temporal gyrus	38	L	6.83	–44 12 –9
Basal ganglia (caudate)		R	6.10	20 6 19
“ “		R	5.37	20 20 8
Thalamus		L	10.22	–8 –34 0
“ “		M	9.57	0 –16 14
**CORRELATION WITH ABILITY TO EXPRESS (IN THE COMPARISON OF EMOTION V NEUTRAL)**				
Inferior Frontal Gyrus	47	R	5.42	40 25 0
Insula (claustrum)		R	5.43	29 –5 17
“ “		R	4.45	28 22 –1
Parahippocampal Gyrus	19	R	4.93	20 –43 –2
“ “	“ “	R	4.30	29 –46 2
Thalamus		R	6.34	23 –13 17
“ “		R	6.21	9 –19 14
“ “		R	5.86	17 –22 14
“ “		L	5.07	–5 –17 4

*Data represent activation foci that survived the probability threshold of p < 0.05 (FWE corrected) and a contiguity threshold of 10 active voxels. Coordinates are given for the “Talairach” stereotactic space (Talairach and Tournoux, 1988). L, left hemisphere; R, right hemisphere; M, midline*.

We also examined which of the brain regions associated with the expression of emotional prosody showed variable activity dependent on participants' ability to express a given emotional tone. This endeavor revealed correlations with activity in the right inferior frontal gyrus, insula, and basal ganglia (Table 2, Figure 3). SPSS was subsequently used to reanalyse and confirm the SPM-generated correlation, between the accuracy with which participants were able to express emotional prosodic contours, and the parameter estimate for the emotional vs. neutral contrast in the inferior frontal gyrus. For this follow-up analysis, the parameter estimates were derived using a 5 mm diameter sphere centered at the peak inferior frontal gyrus activity coordinates indicated in the main analysis of regions whose activity correlated with the ability to express emotional prosody.

## Discussion

In this study, we aimed to make further progress in delineating the functional neuroanatomy of prosodic emotion expression in three ways: Firstly, by incorporating methodological refinements; secondly, by honing in on how the network of brain regions required might differ as a function of positive and negative valence exemplars; and thirdly by determining the parts of the system subject to individual differences in ability. The key findings of our study are that the conjunction analyses delineated common regions of activation for the expression of both angry and happy prosody in the superior temporal gyrus and basal ganglia. Producing a neutral prosodic contour without conveying emotion was also associated with activation in the anterior cingulate, superior temporal gyrus, insula, and basal ganglia. In addition, direct comparisons revealed that expressing angry prosody resulted in greater activation in parts of the basal ganglia and insula compared to happy prosody, whilst expressing happy prosody resulted in greater activation of the anterior cingulate and other parts of the insula and basal ganglia compared to angry prosody. We observed inter-participant variability in the brain regions that support prosodic emotion expression, with activity in the right inferior frontal gyrus and insula correlating with external off-line judgments of the behavioral ability to express emotions prosodically.

### Brain regions recruited for expressing emotions through prosody

Across the expression of anger and happiness, we observed common activation in the superior temporal gyrus and basal ganglia. Data from a number of early lesion-studies suggested that damage to the right-hemisphere homolog of Broca's area impaired the ability to express emotional prosody (Ross, 1981; Ross et al., 1981; Gorelick and Ross, 1987; Nakhutina et al., 2006; Ross and Monnot, 2008). The theory that the organization of prosodic functions in the right-hemisphere mirrors that of propositional language in the left—has been called into question though (Kotz et al., 2003, 2006; Schirmer and Kotz, 2006; Wildgruber et al., 2006; Bruck et al., 2011; Kotz and Paulmann, 2011). If the expression of emotional prosody is also more complex than suggested by the early lesion-studies, perhaps we should not automatically assume activation of the brain regions associated with impaired performance in those early studies. Previous work has used different types of base stimuli to carry the expression of emotions through prosody, ranging from sentences (Pichon and Kell, 2013), through repetitive syllables (Mayer et al., 2002; Aziz-Zadeh et al., 2010), to short pseudowords (Frühholz et al., 2014; Klaas et al., 2015), that may in theory lead to differences in the degree of activation of a given region. The likely complexity of emotional prosody expression is highlighted by inconsistent involvement of the inferior frontal gyrus in its expression across the neuroimaging studies contributing to the literature thus far. Beyond these complexity issues, the impact of individual differences in social cognition also has important theoretical implications, as outlined in the introduction. Being able to infer the thoughts, feelings, and intentions of those around us is indispensable in order to function in a social world. Despite growing interest in social cognition and its neural underpinnings, the factors that contribute to successful mental state attribution remain unclear. Current knowledge is often limited because studies fail to capture individual variability (Deuse et al., 2016). An individual differences dependent neuroanatomical network for the expression of emotional prosody may reflect the necessity to combine multiple functions to successfully convey the target emotion (Valk et al., 2016). For all these reasons, we explored individual differences in the neural system that underpins the expression of prosodic emotions, i.e., we sought to determine whether the network of brain regions used to express emotional prosody, was moderated by individual levels of proficiency in expressing these cues.

Our individual differences aim was operationalized by probing in which brain regions activated during expression of emotional prosody did the ratings of the ability to convey the desired emotional states correlate with the level of activation? Participants who were more able to express emotional prosody on demand would therefore show greater activation in the brain regions thus identified. Whilst research on emotional prosody expression would ideally index participants' abilities online rather than offline, there was little reason to suspect that participants' performance might be unstable over the short time period between offline behavioral assessment and the fMRI session. Nevertheless, the analysis of correlation between level of activation and ability to express emotional prosody could have important implications for neuropsychological studies. Thus, a patient who is poor at expressing prosodic emotions, is likely to be impaired at the neurocognitive level, in the brain regions required to express these cues. Conversely, this same correlation would enable the prediction of expected behavioral impairment, for a patient with known damage to these regions. One of the regions in which such a relationship was observed was the right inferior frontal gyrus. The inferior frontal gyrus is often activated during emotion regulation tasks (Mincic, 2010; Grecucci et al., 2012), and again may be linked to expected performance demands, as those who are better at regulating the desired emotion and display more intense activation of this region may be those who best convey the desired emotion through prosody. There has also been a recent demonstration of a relationship between the level of activation of the inferior frontal gyrus and the intensity used in expressing emotional prosody (Frühholz et al., 2014). This external finding might explain the reason why inferior frontal gyrus activity might correlate with the ability to express appropriate emotional prosody. In the context of our own findings, their demonstration suggests the interpretation that emotional prosody expressed by people who use greater intensity when doing so might be easier to identify for the listener. Indeed, such an interpretation is supported by extant behavioral literature (Chen et al., 2012; Grossman and Tager-Flusberg, 2012). Of course, it may be a limitation of the current study that its participant pool was restricted to highly-educated students based in a university environment. Even though this design feature is in accordance with the other major works on this subject (Aziz-Zadeh et al., 2010; Pichon and Kell, 2013; Frühholz et al., 2014; Klaas et al., 2015), evidence is starting to emerge that in-group and out-group effects may impinge on the comprehension of emotional prosody from speakers (Laukka et al., 2014; Paulmann and Uskul, 2014), thus future studies may seek to broaden the evidence base and sample participants from other educational backgrounds and environments.

The common superior temporal cortex activation we observed across participants, was in an anterior section extending into the superior temporal sulcus, similar to that observed in the preliminary study of Dogil et al. in which participants expressed happiness and sadness through prosody (Dogil et al., 2002). This would not be the first time this region has been suggested as having a role in speech production (Sassa et al., 2007). Anterior superior temporal gyrus/sulcus activity has also been previously observed with various forms of speech comprehension rather than expression, involving semantic processing (Binney et al., 2010; Visser and Lambon Ralph, 2011), accent processing (Hailstone et al., 2012), sensitivity to human voice (Capilla et al., 2013), speech intelligibility (Scott et al., 2000; Friederici et al., 2010; Obleser and Kotz, 2010; Okada et al., 2010), and sensitivity to spectral and temporal features (Obleser et al., 2008). Anterior superior temporal gyrus activity during emotional prosody expression could therefore represent an internal feedback system on aspects of speech related to prosody, particularly vocal qualities of speech (Klaas et al., 2015). Thus, as the data of Aziz-Zadeh et al. suggest, there might be some overlap in the neural systems responsible for expressing and perceiving emotional prosody (Aziz-Zadeh et al., 2010). Importantly, this feedback system cannot be explained away as resulting from the mere act of listening to one's own speech because regional brain activity associated with producing a neutral prosodic contour was controlled for in our analysis. Whilst superior temporal gyrus activity was also observed in the neutral condition, here it was specific to the expression of emotion.

An anterior superior temporal gyrus section was active during execution of emotional prosody in the study by Pichon and Kell (2013). By analyzing the conjunction of regions activated by angry and happy prosodic emotion expression rather than contrasting emotion trials vs. neutral without distinguishing emotion type, we are able not just to confirm the involvement of anterior superior temporal cortex in prosodic emotion expression, but to confirm its overlapping involvement in expressing both a positive and negative emotion. Given that our design was unbiased toward negative vs. positive emotions, the superior temporal gyrus activation we observed may represent a core brain region activated during prosodic emotion expression, regardless of valence. Given that our design did not mix emotional and non-emotional prosody, it is possible that we may also have had increased statistical power to detect activity in the superior temporal gyrus during the expression of emotional prosody in comparison to previous works (Aziz-Zadeh et al., 2010). Given that the anterior temporal lobe activation we observed was in a region sometimes affected by probable susceptibility artifacts (Devlin et al., 2000), it is not necessarily surprising that its involvement is not always picked up in fMRI studies. Activation in this region can also be highly susceptible to experimental “noise” caused by methodological and statistical differences between fMRI studies of speech production (Adank, 2012).

The other key region activated regardless of the specific emotion expressed lay in the basal ganglia, in particular, the caudate. Its activation has previously been observed, although our study could indicate a more general role in expressing prosodic emotions beyond a specific role in expressing happiness (Aziz-Zadeh et al., 2010) or anger (Frühholz et al., 2014; Klaas et al., 2015). Whilst Pichon and Kell only observed striatal activity during preparation for prosodic emotion expression (Pichon and Kell, 2013), our analyses suggest that it may have an important ongoing role in executing emotional prosody. Its involvement in the network of brain regions recruited to express emotional prosody could be interpreted in two ways. First, it could be because of a direct role in expressing prosodic emotions. Whether from a brain lesion or from Parkinson's disease, damage to the basal ganglia typically leads to a monotonous voice devoid of prosodic expressivity and emotion cues (Cancelliere and Kertesz, 1990; Blonder et al., 1995; Schröder et al., 2010a). This direct role could be due to its involvement in timing-related processes (Kotz and Schwartze, 2010), which could establish basic timing patterns from which to formulate emotion-specific patterns of intonation, by integrating dynamically changing speech information such as speech rate, pitch, or amplitude (intensity) variations required for individual emotions (Paulmann and Pell, 2010). The second possibility is that its involvement is indirect, because of its well-evidenced role in the comprehension of prosodic emotions (Mitchell and Bouças, 2009; Schröder et al., 2010a; Bruck et al., 2011; Paulmann et al., 2011; Belyk and Brown, 2014). From these studies that noted its role in emotional prosody comprehension, we can now confirm that the basal ganglia may also be of importance in the expression of emotional prosody.

Adding to prior findings, our study also suggests that as for the inferior frontal gyrus activity we observed, insula activation can be modulated by participants' ability to correctly express happiness and anger through prosody. Other literature shows that insula activation can demonstrate a relationship with emotional intensity (Zaki et al., 2012; Satpute et al., 2013). Although it might require further study, perhaps the greater the activity in the insula, the better someone is at expressing emotions, i.e., the more intense the emotions they can express through prosody. Observing changes in the activity of such regions as patients recover from brain damage affecting the network that normally mediates emotion expression, could be a useful index for research purposes of the transition from monotone speech back to full expressivity. In terms of likely impact on functional outcome, ascertaining the relationship between the ability to express target emotions through prosody, the associated functional neuroanatomy and measures of social function in healthy young adults could further suggest how differences in expression and neural activity map onto such behavioral effects.

### Emotion-specific brain activity

Our paradigm required participants to express anger, happiness and neutrality through prosody. Whilst we do not claim neutrality to be an emotion, it is still a prosodic contour just the same as anger or happiness. In the prior literature, Mayer et al. and Dogil et al. analyzed the expression of happiness and sadness together as a single emotion condition rather than separately (Dogil et al., 2002; Mayer et al., 2002). Pichon and Kell had a design that could have provided rich data on the expression of specific emotions through prosody, including fear, sadness, anger, and happiness (vs. neutrality), but the separate analyses of these emotions were not presented (Pichon and Kell, 2013). In our study, we were able to identify that the expression of angry prosody was associated with activation in the inferior frontal gyrus, superior temporal gyrus, insula, and basal ganglia. The expression of happy prosody was associated with activation of the anterior cingulate, superior temporal gyrus, insula, and basal ganglia. It is, of course, a limitation of the current study that online behavioral recordings were not available for the emotional prosody expression task whilst performed during the fMRI scanning. Therefore, at the time of fMRI data capture, we cannot say for certain which emotion was being expressed through prosody for each trial. Whilst the offline behavioral recordings give a useful indication of each individual's ability to modulate prosody to convey the target emotion, personality-linked dispositional indicators of emotionality may have strengthened these assumptions.

As explained above, it is difficult to compare these data to the results of the few previous studies of prosodic emotion expression. However, the network of regions activated when our participants expressed happy prosody are largely comparable to the valence-linked comparison of happy vs. neutral trials by Aziz-Zadeh et al., and we are able to extend this work to propose the addition of the superior temporal cortex activity (Aziz-Zadeh et al., 2010). The anterior cingulate gyrus, superior temporal gyrus, insula and basal ganglia activation we observed are all regions observed in neuroimaging studies of processing other people's happiness cues (albeit in the facial domain) (Phan et al., 2002; Murphy et al., 2003; Fusar-Poli et al., 2009; Vytal and Hamann, 2010). A more relevant argument can be made in the case of the activations observed in the inferior frontal and superior temporal gyri, insula and basal ganglia when participants expressed angry prosody, as also found by Klaas et al. except for the insula (Klaas et al., 2015), because they have also been associated with the perception of angry prosody (Grandjean et al., 2005; Sander et al., 2005; Quadflieg et al., 2008; Hoekert et al., 2010; Frühholz and Grandjean, 2012; Mothes-Lasch et al., 2012). The combination of evidence from these pre-existing studies and our own data may again lead one to conclude overlapping networks for perceiving and expressing positive and negative emotions. However, there are also pockets of evidence that the anterior cingulate gyrus, superior temporal gyrus, insula, and basal ganglia are involved in the facial expression of happiness, not just its perception (Lee et al., 2006; Kühn et al., 2011; Pohl et al., 2013). If involved in expression happiness through both prosody and facial expressions, these brain regions may have a supramodal role in expressing emotion cues like that which exists for perceiving emotion cues (Vuilleumier and Pourtois, 2007; Park et al., 2010; Peelen et al., 2010; Klasen et al., 2011). There is a lack of evidence as to whether the regions involved in expressing angry prosody overlap with the brain regions involved in expressing anger through facial expressions though.

A new interpretation that we think also deserves consideration comes from evidence that the basal ganglia and limbic brain structures are involved in feeling or being happy (Phan et al., 2002; Kringelbach and Berridge, 2009). Although our participants were required to act the designated emotions rather than portray them naturally, it seems from our data that there may potentially have been an automatic mood induction effect (Siemer, 2005; Rochman et al., 2008). This explanation also fits well with our data on expressing anger through prosody, since activation of the inferior frontal gyrus, insula, and thalamus have all been associated with feeling anger (Kimbrell et al., 1999; Denson et al., 2009; Fabiansson et al., 2012). This hypothesis could quite easily be tested in the future by employing explicit mood induction procedures to invoke a happy or angry experiential state and then whilst in that state asking participants to express the corresponding emotions. Whilst the act of preparing to express emotional prosody has been speculated as an induction phase, the study concerned did not explicitly assess mood state (Pichon and Kell, 2013).

As well as examining the brain regions involved in expressing anger and happiness through prosody separately, we directly compared the two whilst accounting for differences in performance accuracy between the conditions. It is well accepted in facial emotion research that beyond the core processing network, additional brain regions are involved in expressing specific emotions (Chakrabarti et al., 2006; Fusar-Poli et al., 2009; Hamann, 2012). There is preliminary evidence that this may also be the case for emotional prosody (Ethofer et al., 2008; Jacob et al., 2012; Kotz et al., 2013b). Although the two separate valence-related analyses of happy and angry prosody expression seemed to suggest that inferior frontal gyrus activity was greater for angry prosody expression and that anterior cingulate activity seemed to be greater for happy prosody expression than for angry prosody expression, only the latter was statistically significant. Therefore, it is not certain whether inferior frontal gyrus activity during the expression of prosody is emotion-specific as it was for individual differences in performance accuracy. That a major emotion-related brain region such as the anterior cingulate should show a greater neural response to anger expression than to happiness is perhaps not surprising given the evidence that our brains are evolutionally predisposed to processing those emotions associated with threat (Vuilleumier and Schwartz, 2001; Guastella et al., 2009). We also observed differential emotion-dependent activations within the insula and basal ganglia. Thus, the expression of angry and happy prosody both activated the basal ganglia and insula, but the foci of these activations were in spatially separate parts of these structures. There are suggestions that the activation in the caudate and/or putamen whilst processing prosodic information may be emotion-specific (Kotz et al., 2013a), however, there is not yet enough research to judge the reliability of spatially separate emotion-specific activations within the basal ganglia and insula.

Finally, our inclusion of a jaw movement condition allowed us to also examine which brain regions were recruited for expressing neutral prosodic contours, not just emotional contours. Knowing the brain regions associated with expressing neutral prosody would allow clinicians to distinguish between patient groups for which expressing a certain emotion is compromised, and those groups who have difficulty in expressing prosodic contours of any type. In the comparison of neutral prosody and jaw movement, activations observed in the basal ganglia and superior temporal gyrus are especially interesting. Whilst data from the analysis of regions involved in expressing emotional prosody irrespective of the specific emotion observed basal ganglia involvement, our additional data on expressing neutrality suggest a more fundamental role for this structure in producing intonation. Whilst the basal ganglia was activated by expressing both neutral and emotional prosody, the activation observed in the case of emotional prosody controlled for those brain regions already involved in the production of neutral prosody. Therefore, it has both a specific role in producing emotional prosodic contours, and a more general role in producing prosody without emotion. This finding is intuitive given the generic difficulties experienced by patients with basal ganglia pathology (e.g., Parkinson's disease) in producing prosodic contours (Schröder et al., 2010b; Martens et al., 2011). In relation to the superior temporal gyrus activation observed expressing neutral prosody, the cluster bordered onto the superior temporal sulcus. This region has been identified as having a key role in aspects of pitch processing (Griffiths, 2003; Stewart et al., 2008). Its role in producing pitch contours devoid of emotional connotation could therefore indicate a self-monitoring process as people express prosody, to ensure that the pitch pattern of their speech at any one point in time is appropriate.

## Conclusions

In summary, we conclude that the superior temporal gyrus and basal ganglia may be involved in expressing emotional prosody irrespective of the specific emotion. Inferior frontal gyrus activity may be more variable, and might relate to the participants sampled since its activity correlated with participants' ability to express the target prosodic emotions. In addition to the core network, the location of other activation foci may depend on emotion valence, as direct comparison of the functional neuroanatomy associated with expressing angry and happy prosody established that expression of angry prosody was associated with greater activity in the inferior frontal gyrus, whereas expression of happy prosody was associated with greater activity in the anterior cingulate.

## Author contributions

RM conceived and designed the study, performed the paradigm programming, provided technical assistance, assisted with data collection, analyzed the results, and wrote the manuscript. AJ and MS assisted with data collection. SK assisted with the study design and with writing the manuscript.

## Funding

This study was funded by Durham University.

### Conflict of interest statement

The authors declare that the research was conducted in the absence of any commercial or financial relationships that could be construed as a potential conflict of interest.
