# Supplementary material for: Recruitment of Language-, Emotion- and Speech-Timing Associated Brain Regions for Expressing Emotional Prosody: Investigation of Functional Neuroanatomy with fMRI
Source: Front Hum Neurosci. 2016 Oct 18;10:518. doi: 10.3389/fnhum.2016.00518 (PMC5067951; doi:10.3389/fnhum.2016.00518)
Supplement: Supplementary file 1 [file DataSheet1.DOCX]

**Supplementary Materials**

**Nonsense words from the prosody expression task**

The stimuli which formed the basis of the emotional prosody expression task used inside and outside the MRI scanner consisted of pronounceable nonsense words as follows:

*moy*

*dask*

*tarm*

*broath*

*spoaker*

*mintle*

*orase*

*clath*

*slem*

*tosk*

*crob*

*crume*

*claf*

*bradge*

*hant*

*nid*

*sheve*

*nuck*

*plin*

*polace*

*im*

*narmal*

*borth*

*thomb*

*shap*

*tham*

*cheld*

*nug*

*wutch*

*mep*

*stip*

*smill*

*papper*

*kess*

*smike*

*thomb*

*shap*

*tham*

*cheld*

*nug*

*wutch*

*wab*

*narse*

*shrib*

*lettle*

*clawn*

*snew*

*namber*

*tib*

*prace*

*dillar*

*moke*

*stond*

*lote*

*skall*

*nane*

*selid*

*fulse*

*fut*

*crade*

*fest*

*wes*

*wenter*

*eags*

*whote*

*drenk*

*grice*

*ent*

*jonk*

*het*

*af*

*sammer*

*cless*

*soath*

*wode*

*bist*

*misic*

*igg*

*dod*

*boak*

*hote*

*luds*

*drap*

*ged*

*brish*

*naver*

*sester*
